# Molecular Insights into the Potential of Extracellular Vesicles Released from Mesenchymal Stem Cells and Other Cells in the Therapy of Hematologic Malignancies

**DOI:** 10.1155/2021/6633386

**Published:** 2021-02-17

**Authors:** Suliman A. Alsagaby

**Affiliations:** Department of Medical Laboratories Sciences, College of Applied Medical Sciences, Majmaah University, Majmaah 11932, PO Box 1712, Saudi Arabia

## Abstract

Hematologic cancer encompasses the heterogeneous group of neoplasms that affect different stages of blood cell linages. Despite the significant improvements made in the new modalities of anticancer therapy, many forms of blood cancer remain untreatable, putting the afflicted patients at high risk of death. Therefore, there has been an urgent need for novel therapy to improve the clinical outcomes of patients with blood cancer. Mesenchymal stem cell-derived extracellular vesicles (MSC-EVs) have been reported to possess an anticancer activity. This review discusses (i) the therapeutic potential of MSC-EVs against blood cancer, (ii) the possibility of using EVs from sources other than MSCs as a mean for blood cancer vaccination and drug delivery, and (iii) areas to be optimized for MSC-EV-based clinical application on blood malignancies.

## 1. Introduction

Blood cancer comprises a wide range of tumors that originate from multiple stages of myeloid or lymphoid linages [1]. Common forms of blood neoplasms include acute myeloid leukemia (AML), chronic myeloid leukemia (CML), acute lymphoblastic leukemia (ALL), chronic lymphocytic leukemia (CLL), multiple myeloma (MM), and lymphoma [2]. Chromosomal abnormalities and gene mutations have been heavily implicated in the pathogenesis and prognosis of hematologic malignancies [3–6]. For instance, the fusion of breakpoint cluster region-tyrosine-protein kinase with tyrosine-protein kinase ABL (*BCR*-*ABL*) seen in t (9; 22) is a driving mutation and characteristic of CML [7]. The deletion of cellular tumor antigen p53 (*TP53)* [del (17p)] underpins chemotherapy resistance in CLL [8]. The overexpression of *cyclin D1* in *t* (11; 14) was shown to drive the growth of MM and mental cell lymphoma [9]. Similarly, the overproduction of apoptosis regulator Bcl-2 (*BCL2*) as a result of *t* (14; 18) is a hallmark and a driving mutation of follicular lymphoma [10]. Collectively, chromosomal alterations and gene mutations play a major role in the initiation and progression of blood neoplasms.

Although cancer is an age-associated disease, blood cancer is seen in adults as well as in children [11]. According to the Surveillance, Epidemiology, and End Results (SEER) Program, 137,770 new cases of leukemia and lymphoma are estimated to be recorded in the USA by the end of 2020. Despite the advances made in cancer therapy, many forms of blood cancer remain incurable [12–15]. Furthermore, chemotherapy, which is still the front-line treatment for many blood neoplasms, causes serious side effects, and drug resistance is inevitable in some cases. The five-year overall survival (5-year OS) of patients with blood cancer remains unsatisfactory; according to SEER, the 5-year OS of patients with leukemia and lymphoma was 63% and 72%, respectively. Therefore, there has been a great need for new modalities of anticancer therapy to improve the clinical outcomes of patients diagnosed with hematologic neoplasms. Interestingly, extracellular vesicles (EVs) released from mesenchymal stem cells (MSCs) have emerged as a promising mean for therapy of various diseases, such as cancer, kidney injury, liver fibrosis, traumatic brain injury, acute spinal cord injury, and myocardial infraction [16]. The present work discusses (i) the therapeutic potential of MSC-derived EVs on blood cancer, (ii) the potential of using EVs from cells other than MSCs for blood cancer vaccination and drug delivery, and (iii) areas to be optimized for MSC-EV-based clinical application on blood malignancies.

## 2. Extracellular Vesicles

**Figure 1:**
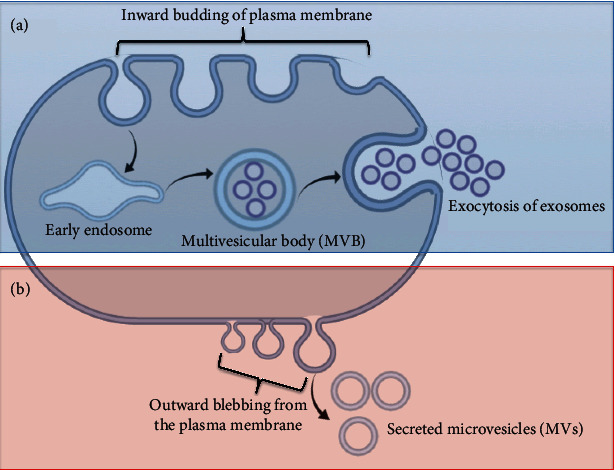
Biogenesis of extracellular vesicles (EVs). Section (a) describes the biosynthesis of exosomes, and section (b) shows the generation of microvesicles (MVs).

Extracellular vesicles (EVs) are small, bilayer, membrane-enclosed vesicles that are secreted from nearly all types of cells. EVs vary from each other by their biosynthesis, size, content, and surface markers [17]. Exosomes and microvesicles (MVs) are two common examples of EVs, with differences between them being reported on the bases of biogenesis, composition and size [18]. Exosomes are smaller than MVs with a size ranging from 40 to 100 nm in diameter and are generated by inward budding of the plasma membrane to form an early endosome that maturates into late circular endosome enclosing intraluminal vesicles (ILVs). Multivesicular bodies (MVBs) describe late rounded endosomes filled with large number of ILVs. MVBs fuse with the plasma membrane and release their content by exocytosis to the extracellular space. At this point, the secreted ILVs are known as exosomes (Figure 1(a)). The generation of exosomes is tightly controlled by a range of mechanisms including elements of the endosomal sorting complex required for transport (ESCRT), Rab proteins, tumor protein p53/tumor suppressor-activated pathway-6 pathway, ceramide, and neutral sphingomyelinase [19]. In contrast, MVs are bigger in size (50-1000 nm in diameter) and are created and released by outward blebbing from the plasma membrane (Figure 1(b)) [20]. Given the nature of the production of MVs, the composition of their plasma membrane shows high resemblance with that of the parent cells. Noticeably, the surface of MVs is enriched with integrins, glycoprotein Ib (GPIb), and P-selectin. The molecular content of EVs is further discussed in a later subsection. The old historical view of EVs states that they are generated and secreted from cells to eliminate cellular waste. However, further research showed that EVs are very important for cellular communications [21]. EVs circulate in different body fluids, such as blood, breast milk, and urine, in search of their target cells that they recognize via specific receptors/ligands. Once the target cells are reached, EVs exert their impact either by receptor-ligand binding of EVs with recipient cells or by delivering their content into the target cells through endocytosis, phagocytosis, pinocytosis, or membrane fusion [22]. The molecular content of EVs includes mRNAs, microRNAs, proteins, and soluble factors that alter the behavior of target cells [23]. EVs affect various biological processes—such as proliferation, apoptosis, migration, and angiogenesis—of recipient cells [24].

### 2.1. Isolation of Extracellular Vesicles

Extensive research has been conducted to describe and characterize various methods and technologies for the isolation of EVs. It appears that there is no golden method of EV extraction that fits all proposes, as each method has its advantages and drawbacks [25]. Therefore, understanding the strengths and weaknesses of each method is essential for selecting the suitable procedure that meets the goal of using EVs. Ultracentrifugation is the most commonly utilized method, where differential centrifugation is first conducted to sediment cells, cellular debris, apoptotic bodies, and aggregates of biopolymers. Next, EVs contained in the resulting supernatant are isolated by ultracentrifugation at >100,000 × g (100,000–200,000 × g) for 2 hours. Multiple washing of the isolated EVs followed by ultracentrifugation is recommended to reduce non-EV proteins [26]. This method allows the isolation of EVs from large volume samples, and it does not require additional of chemicals, but it uses expensive equipment (ultraspeed centrifuge), has low reproducibility, and provides low RNA yield with non-EV impurity [27]. Importantly, the ultragravity forces used in ultraspeed centrifuge may cause damage to the EVs. Density gradient ultracentrifugation has gained attraction as it overcomes the impurity of EVs isolated by ultracentrifugation [28]. EVs are separated in a density gradient medium, such as sucrose, using a long ultracentrifugation step that consumes 250 min to 48 hours. The RNA yield in the two methods (ultracentrifugation and density gradient ultracentrifugation) was reported to be similar [29]. Another method that was reported to preserve the integrity of EVs and reward highly pure preparations is size exclusion chromatography (SEC) [30]. However, in this method, there is limitation in the number and volume samples that can be processed (one sample at a time). Ultrafiltration is also a common method, where membrane with nano-sized pores (diameter of 0.8–0.1 *μ*m) is used to retain EVs with size ranging from 800 to 100 nm [31]. This method is simple and time-effective (consumes 130 min), allowing more samples to be processed. It also yields pure preparations with no need for addition of chemicals that may affect the EVs. Ultrafiltration can be used in combination with ultracentrifugation or SEC to increase the purity of the isolated EVs. Other less common methods include column-based affinity, Annexin A5-coated magnetic beads, and immunoprecipitation [27].

### 2.2. Mesenchymal Stem Cells and Hemopoiesis

Mesenchymal stem cells (MSCs) are nonhematopoietic cells that are characterized by (i) the surface expression of CD73, CD90, and CD105 with absence of CD19, CD14, CD34, CD45, and human leukocyte antigen-DR (HLA-DR); (ii) adhesion to plastic; and (iii) the ability to differentiate in vitro under specific conditions into different types of cells, such as adipocytes, chondrocyte, and osteocytes [32]. MSCs were reported to be isolatable from multiple sources like bone marrow, fat tissue, umbilical cord, dental pulp, placenta, and synovial fluid [33]. MSCs are indispensable components of the bone morrow (BM) microenvironment and play essential roles in hemopoiesis [33]. In the BM microenvironment, stromal cells—which consist of MSCs, adipocyte fibroblast osteoblast, and endothelial cells—are the constituents of hemopoietic niche, where hematopoietic stem cells (HSCs) reside. MSCs and other stromal cells in the hemopoietic niche provide prosurvival and stemness signals needed to protect HSCs from cell death and differentiation. As a result, HSCs are maintained alive with the ability to undergo self-renewal [34, 35]. A typical example of the crosstalk between MSCs and HSCs is the engagement of stem cell factor (SCF) on MSCs with its receptor (SCFR) on HSCs to promote the maintenance and self-renewal of the latter [36]. In fact, deletion of *SCF* led to depletion of HSCs in a mouse model [36]. Similarly, the interaction between C-X-C motif chemokine ligand 12 (CXCL12) released from MSCs and C-X-C motif chemokine 4 receptor (CXC4R) on HSCs is necessary to maintain HSCs [37, 38]. Wnt signaling was reported to promote self-renewal and survival of HSCs [39]. Agrin, which is proteoglycan involved in the neuromuscular junction, was found to be expressed by MSCs and to induce proliferation and survival of HSCs [40]. Integrin-beta1-dependent contact between MSCs and HSCs was shown to regulate the self-renewal capacity of HSCs [41]. Taken together, normally functioning MSCs are essential for successful hemopoiesis.

### 2.3. Molecular Composition of MSC-EVs

The cellular origin of EVs has an impact on the molecular composition of released EVs. For instance, in addition to the known markers of exosomes (CD63, CD9, CD81, ALG-2-interacting protein X (Alix), lysosome-associated membrane glycoprotein 1 (LAMP1), and heat shock protein 7 (HSP7)), exosomes released from MSCs exhibit the typical markers of MSCs (CD29, CD73, CD90, CD44, and CD105), reflecting their cellular origin [42]. Three lipid species (diacyleglyscerole, sphingomyelin, and ceramides) are commonly reported in the membrane lipid of MSC-EVs [43]. Interestingly, along with the structural roles of these lipids, they have also been implicated in signal transduction, cell cycle arrest, tumor suppression, and apoptosis [44]. Applying omic approaches enabled researcher to deeply characterize the molecular content of MSC-EVs. In this context, Haraszti et al. explored the proteome and lipidome content of exosomes and MVs derived from MSCs [45]. The authors reported 972 proteins in the exosomes and 1874 proteins in the MVs. Translation, glycolysis GTPase activity, and cell motion were pathways that were significantly enriched by the common proteins in the exosomes and MVs. Extracellular matrix, binding (receptor, heparin, phospholipid, integrin) immune response, and cell adhesion were enriched by proteins specific to exosomes. In contrast, proteins that were detected only in MVs enriched mitochondria, endoplasmic reticulum, and proteasome. These data highlight the differential composition of exosomes and MVs. The lipidomic work also reported 1961 lipid species that differed from each other by head group, number, length, and saturation of fatty acid tails. Differential lipidome was reported in the exosomes and MVs. For instance, acyl carnitines, lysophosphatidyl cholines, cholesterol esters, ceramides, and sphingomyelins showed higher concentration in MVs compared with exosomes. In contrast, free fatty acids, cardiolipins, glycolipid, and lysophosphatidyl serines exhibited increased concentration in exosomes compared with MVs. Another study explored the proteome of exosomes released from three different sources (umbilical cord (UC) MSCs, bone marrow (BM) MSCs, and adipose tissue (AT) MSCs) that reported 1014 proteins of which 37 protein, 23 proteins, and 341 proteins were specific to UC-MSC-exosomes, AT-MSC-exosomes, and BM-MSC-exosomes, respectively [46]. These data may reflect the impact of exosome source on the exosome content and perhaps function. In contrast to the exosomes of UC and AT, those released from BM highly expressed proteins that have been implicated in transcription activation, integrin-mediated signaling, monocyte activation, innate immune, hypoxia, and tubulin binding. In the context of nucleic acids, Baglio et al. compared the RNA pool in MSCs and in their exosomes and reported a striking RNA signature being associated with exosomes [47]. The most abundant class of RNA in MSCs was small nuclear RNA (snoRNA) followed by miRNA. However, transfer RNA and repeat RNA dominated the exosome content of RNA. Noticeably, the exosome composition of miRNA was small (2-5% of the total of small RNA), but was dominated (43-59% of miRNA) by five miRNAs (miR-143-3p, miR-10b-5p, miR-486-5p, miR-22-3p, and miR-21-5p) that were implicated in the regulation of cell cycle, immune response, and cell migration. This finding indicated a selective incorporation of miRNAs into exosomes perhaps to fulfill the specific function. Ferguson et al. characterized the MSC-exosome content of miRNA; the most abundant miRNAs (*n* = 23) were predicted to target 5481 genes with high stringency. These genes constitute pathways like Wnt signaling, profibrotic signaling via transforming growth factor beta (TGF-*β*) and platelet-derived growth factor (PDGF), proliferation, and apoptosis [48].

### 2.4. Normal MSC-EV-Based Therapy

Given the roles that normal MSCs play in hematopoiesis, it has been suggested that EVs secreted from normal MSCs may restore normal hematopoiesis in patients with blood cancer. In line with this view, in vivo findings reported by Roccaro et al. showed that tumor growth and metastasis of multiple myeloma (MM) in mice inoculated with the MM cell line (MM.1S) and exosomes released from primary MM BM-MSCs were high compared with that seen in mice administrated with MM cells and exosomes derived from normal BM-MSCs [49]. A later study attempted to explain the opposite impact of MM-BM-MSC-EVs and normal BM-MCS-EVs on the growth and metastasis of MM [50]. The study showed that in contrast to BM-MSC-MVs from MM patients, normal BM-MSC-MVs reduced the proliferation, migration, and survival of five MM cell lines (U266, ARP-1, MM.1S, OPM-2, and RPMI 8226). The authors attributed the differential effect of BM-MSC-MVs from MM patients and healthy donors on MM cells to the less adequacy of normal BM-MSC-EVs compared with MM BM-MSC-EVs to increase the expression and/or phosphorylation of mitogen-activated protein kinases (MAPKs: extracellular signal-regulated kinase 1/2 (ERK1/2) and c-Jun N-terminal kinase (JNK)), translation initiation (TI) factors (eukaryotic translation initiation factor 4GI (eIF4GI) and eukaryotic translation initiation factor 4E (eIF4E)), TI regulator (target of rapamycin kinase (TOR), MAP kinase-interacting serine/threonine-protein kinase 1/2 (MNK1/2) and eukaryotic translation initiation factor 4E-binding protein (4EBP)) and oncogenes (nuclear factor kappaB (NF*κ*B), mothers against decapentaplegic homolog 5 (SMAD5), cyclin D, hypoxia-induced factor 1 alpha (HIF1*α*), and transcriptional effector cMyc (cMyc)). A further clarification of the distinct influence of MM BM-MSC-EVs and normal BM-MSC-EVs came from a study that characterized the content of EVs from the two sources [49]. Interestingly, the levels of oncoproteins, cytokines, and adhesion molecules were greater in MM BM-MSC-EVs compared with normal BM-MSC-EVs. In contrast to MM BM-MSC-EVs, BM-MSC-EVs from healthy donors exhibited low levels of interleukin6 (IL6), C-C motif chemokine 2 (CCL2), and fibronectin, as well as high levels of miR15a. CCL2 was previously shown to be in vitro and in vivo essential for the growth of MM [51, 52], and 15a miR was reported to inhibit the proliferation and induces apoptosis in MM cells through targeting AKT serine/threonine-protein-kinase (AKT3), ribosomal-protein-S6, MAP-kinases, and NF-*κ*B-activator MAP3KIP3 [53]. Collectively, normal BM-MSC-EVs appeared to hold promise for MM therapy; therefore, further work is to be conducted in order to clinically employ them for the treatment of patients with MM.

In the context of the influence of donor age on the therapeutic potential of normal BM-MSC-EVs, young donor-derived BM-MSC-exosomes were found to be more competent to inhibit MM-induced angiogenesis compared with BM-MSC-exosomes from old donors [54]. The exosomal content of miRs in young and old donors was shown to possess different signatures; 24 miRs were upregulated, and 12 miRs were downregulated in BM-MSC-exosomes from young donors compared with those from old donors. Interestingly, the greater therapeutic potential of young donor-derived BM-MSC-exosomes attributed, at least partially, to the increased level of miR430 that blocks angiogenesis through the hepatocyte growth factor/hepatocyte growth factor receptor (HGF/HGFR) signaling pathway in endothelial cells [54]. The reduced capability of BM-MSC-exosomes from old people to inhibit MM-induced angiogenesis may explain the association of MM with age [55]. These data may direct the extraction of BM-MSC-exosomes to be limited to young donors as they are better than old donors in providing highly competent BM-MSC-exosomes.

Unlike BM-MSC-EVs of healthy people, BM-MSC-EVs derived from patients with myelodysplastic syndrome (MDS) appeared to play roles in the deregulation of hematopoiesis. Muntión et al. reported increased survival and clonogenicity of hematopoietic progenitor cells CD34+ postincubation with MDS BM-MSC-EVs, but not with BM-MSC-EVs from healthy donors [56]. The researchers studied the exosomal content of miRs and found higher levels of miR132, miR136, miR15a, miR10a, and miR198 in BM-MSC-exosomes from MDS patients compared with healthy donors. Interestingly, miR10a and miR15a are overexpressed hematopoietic cells from MDS patients and were also implicated in the pathogenesis of the disease [57, 58]. Noticeably, the incorporation of MDS BM-MSC-exosomes into hematopoietic progenitors increases the levels of miR10a and miR15a in the later, hence contributing to the development of MDS. [56] In contrast, the incorporation of BM-MSC-exosomes from healthy donors did not elevate the expression of miR10a and miR15a, thus giving a possible explanation of why normal BM-MSC-exosomes did not contribute to the deregulation of hematopoiesis seen in MDS patients [52]. Taken together, normal BM-MSC-exosomes may have the potential to restore normal hemopoiesis in MDS patients.

In acute myeloid leukemia (AML), miR155 and miR375 are independent risk factors of disease recurrence [59]. Interestingly, the exosomal content of these two miRs in relation to parent BM-MSCs was shown to be greater in AML patients compared with healthy donors [60]. Furthermore, BM-MSC-exosomes from AML patients showed a higher level of epidermal growth factor (EGF) compared with those isolated from healthy donors [60]. Interestingly, the expression of epidermal growth factor receptor (EGFR) on AML cells was shown to strongly associate with poor prognosis of the disease, proposing a pathological involvement of EGF/EGFR signaling in AML [61]. These data suggest that unlike normal BM-MSC-exosomes, AML BM-MSC-exosomes support the growth of AML. In line with this view, BM-MSC-exosomes from AML patients, but not from healthy donors, were shown to protect AML cells from chemotherapy (fms-like tyrosine kinase 3 (FLT3) inhibitor AC220) [60]. These data rationalize a possible utility of normal BM-MSC-exosomes in the treatment of AML, especially in patients with resistance to chemotherapy.

In patients with blood malignancies, the application of allogeneic hematopoietic stem cell transplantation (allo-HSCT) has earned wide acceptance due to its curative capability [62]. Suppressed immune reactivity of allo-HSCT recipients, complete clearance of tumor cells in patients, and protection against microbial infections are important factors that increase the success rate of allo-HSCT-based therapy [63]. Low tolerance of the host immune system to allo-HSCT causes an induction of T-cell-based immune response against the transplant, which can lead to engraftment failure; this is seen in the graft versus host disease (GVHD) [64]. Patients who develop GVHD undergo immunosuppressant-based therapy using steroids. However, a substantial proportion of patients shows resistance to the treatment; so, they are considered at high risk of death due to GVDH or its complications [65].

As discussed earlier, MSCs are essential components of the hematopoiesis microenvironment; in fact, MSCs heavily contribute to normal hematopoiesis through their impact on the survival, proliferation, and differentiation of HSCs [66]. Therefore, the systemic infusion of MSCs from BM or UC has been used to improve the clinical outcomes of allo-HSCT. The favored impact of MSCs on allo-HSCT was attributed to the capability of MSCs to modulate the immune reactivity of host and to promote allo-HSCT engraftment [67–71]. Furthermore, in steroid refractory GVDH postallo-HSCT, BM-MSC infusion has also been shown to be a safe treatment option to alleviate the disease burden and improve patients' survival [72–74].

In consistence with roles of MSCs in the improvement of clinical outcomes of allo-HSCT and GVHD, BM-MSC-EVs were reported to enhance the viability and to limit the differentiation of UCHSCs [75]. Noticeably, in vivo coadministration of BM-MSC-EVs with UCHSCs enhanced the migration capacity of the latter from peripheral blood to BM and supported the formation of hemopoietic niche, pointing out the desired impact of BM-MSC-EVs in the engraftment of UCHSCs [75]. Molecular findings provided explanation for these observations: UCHSCs increased the expression of 103 genes and reduced the expression of 100 genes posttreatment with BM-MSC-EVs. The upregulated genes were implicated in cell movement and cell growth, whereas the downregulated genes were involved in cell death. Interestingly, genes that promote cell migration, such as protein L1b (L1b), granulocyte-macrophage colony-stimulating factor (CSF2), C-C motif chemokine 3 (CCL3), endothelial transcription factor GATA-2 (GATA2), and CXCR4, were among the highly expressed genes in UCHSCs. The authors further studied the mechanism by which the BM-MSC-EVs exerted their influence on UCHSCs and reported increased levels of miRs in the EVs whose target genes were downregulated in UCHSCs (miR-3168/LYZ, miR-27b-3p/ZFP36, miR21-5p/ANXA1) [75]. This result indicated that the altered transcriptome of UCHSCs posttreatment with BM-MSC-EVs is driven, at least partially, by the EV content of miRs.

In vivo experiments linked the amelioration of acute GVHD by BM-MSC-exosomes, but not exosomes from normal human dermal fibroblasts (NHDFs) to the ability of the former to suppress T-helper cells and T-cytotoxic cells [76]. Furthermore, the BM-MSC-exosomes inhibited the differentiation of naïve T-cells to effector cells as evidenced by the reversed ratio of CD62L^–^CD44^+^/CD62L^+^ CD44^–^. The BM-MSC-exosomes also preserved naïve regulatory T-cells (Treg); the exosomes repressed the stimulation of CD45RA^+^ Foxp3^low^ naïve Treg to CD45RA^–^ Foxp3^high^ effector Treg. The differential profile of miRs in BM-MSC-exosomes and NHDF-exosomes proposed an explanation for the immunomodulatory roles of BM-MSC-exosomes; miRs that target mRNAs implicated in cellular proliferation, T-cell receptor (TCR) signaling, and GVHD were overexpressed in BM-MSC-exosomes compared with NHDF-exosomes.

In a mouse model of allo-HSCT, human UC-MSC-EVs alleviated the burden of acute GVHD and increased the survival rate of recipient mice [77]. The underpinning mechanism appeared to be the ability of UC-MSC-EVs to elevate the ration of Th cells/T-cytotoxic cells along with the serum level of IL10 and decrease the serum content of interleukin 2 (IL-2), tumor necrosis alpha (TNF-*α*), and tumor necrosis gamma (IFN-*γ*). In the chronic GVHD mice model, exosomes derived from BM-MSCs, but not fibroblasts, were able to lighten the symptoms of the disease [78]. The immunosuppression activity of BM-MSC-exosomes was clearly manifested in their ability to repress the activation and infiltration of CD4 T-cells in the lung tissue. BM-MSC-exosomes induced Treg and inhibited the activation and migration of pathogenic T-cells (Th17) [78], which have been heavily implicated in the pathogenesis of chronic GVHD [79]. In vivo experiments also showed reduced production of Th17 proinflammatory cytokines—such as interleukin 17A (IL-17A), interleukin 21 (IL-21), interleukin 22 (IL-22), and IL-2, caused by MSC-exosomes [78].

Kordelas et al. examined the curative potential of BM-MSC-exosomes in patients with steroid-resistant acute GVHD following allo-HSCT and reported a significant decrease of the disease burden [80]. Interestingly, the authors found increased levels of anti-inflammatory molecules—such as IL10, transforming growth factor beta (TGF-beta) and human leukocyte antigen G (HLAG) in the BM-MSC-exosomes that coincided with the reduced levels of proinflammatory cytokines, including IL6, IL17a, IL21, TNF-*α*, and IFN-*γ* and proapoptosis protein-like soluble FAS ligand. Consistently, a significant decrease in the production of proinflammatory cytokines from patients' BPMCs was found postadministration with BM-MSC-exosomes. Noticeably, the immunosuppression capability of the BM-MSC-exosomes varied significantly between the donors. Of the four donors, one had higher levels of IL10 and TGF beta and lower levels of IFN-*γ*. These findings highlight three points: (i) BM-MSC-exosomes are clinically applicable for the treatment of allo-HSCT-induced steroid-resistant acute GVHD, (ii) the desired effect of BM-MSC-exosomes on GVHD was attributed to the ability of the exosomes to suppress the immunoreaction of host towards the graft, and (iii) evaluating the immunosuppression potential of BM-MSC-exosomes extracted from multiple donors is justified to select the most potent source for clinical application.

## 3. Exosome-Based Vaccine

The content of EVs is derived from their cellular source; hence, EVs cargo can be used to indicate their cellular origin. This finding gave a rationale for using cancer exosomes to direct the immune system to fight cancer cells. The concept of exosome-based vaccine evolves around the idea that cancer-derived exosomes harboring tumor-specific protein is internalized by dendritic cells (DCs) and processed to be presented on their surface in order to activate naïve T-cells [81]. This in turn initiates an immune response specific to tumor cells. Following the same concept, Shen et al. reported that the incorporation of exosomes secreted from acute myeloid leukemia (AML) cells into pulsed dendritic cells (DC) induced cytotoxic T-lymphocytes (CTLs) immunity against AML cells, which killed the leukemic cells [82]. The same notion was applied on chronic myeloid leukemia (CML), where CML-exosomes were incorporated into DCs that initiated CTL-based immunity against CML cells [83]. Similar findings were reported using an in vivo setting; mice administrated with DCs containing CML-exosomes were immune-protected against the development of CML postadministration with CML cells [83]. Interestingly, manipulation of the content of tumor-derived exosomes was shown to support the efficacy of exosome-based cancer vaccine. In an in vivo setting, the forced increased expression of IL2, by transfection, in tumor exosomes showed better immunogenicity against lymphoma cells compared with nontransfected tumor exosomes [84]. Similar findings were observed using tumor exosomes manipulated to overexpress IL18 [85]. Given the roles of transforming growth factor-*β*1 (TGF-*β*1) in immunosuppression, it is employed by tumor cells for immunoescape [86]. Therefore, silenced TGF-*β*1 leukemic exosomes were shown in vivo to be more potent than leukemic exosomes expressing TGF-*β*1 to trigger CTL-based immunity against lymphocytic leukemia [87]. Overall, these data demonstrated a promising potential of exosome-based vaccine in tackling hematologic neoplasms. However, further work is needed to investigate the clinical utility of an exosome-based vaccine.

### 3.1. Targeting EVs

Given the supportive impact of EVs released from the tumor microenvironment, including BM-MSCs, on the growth, survival, and metastasis of blood malignancies, targeting such EVs seems justified for tumor therapy. The protumor role of these EVs was proposed to be targetable by blocking their biogenesis, release, or uptake. Koch et al. blocked exosome biogenesis using indomethacin and reported increased sensitivity of lymphoma cells to chemotherapy [88]. Javidi-Sharifi et al. also reported that inhibition or silencing of fibroblast growth factor receptor (FGFR) blocked the secretion of exosomes, which in turn abolished the protective impact of the tumor microenvironment on leukemia cells [89]. Similarly, inhibition of exosomes release by dimethyl amiloride (DMA) was found to sensitize lymphoma cells (EL4 cell line) to chemotherapy in mice [90]. Depletion of exosomes seems to be an attractive strategy to increase chemotherapy efficacy [91]. MM-derived exosomes were shown to be incorporated into BM-MSCs, promoting their survival and proliferation [92]. Using inhibitor (heparin) that interferes with endocytosis pathways reduced the uptake of MM-exosomes by BM-MSCs, which antagonized the positive impact of the former on the later [92]. Targeting exosome shedding from AML stem cells by increasing the expression of miR-34-5p showed antitumor activity against AML cells in vitro and in vivo [93]. Exosomes secreted from B-cell lymphoma were shown to carry CD20, which was found to consume the anti-CD20 monoclonal antibody rituximab used for the disease therapy. In vitro removal of exosomes increased the cytolytic activity of rituximab against lymphoma cells [94]. It is important to mention that unselective targeting of EVs is a double-edged sword, because it does not distinguish between “pathological” EVs, the targeting of which is beneficial, and the “physiological” EVs, which are necessary for normal cellular communication. Therefore, development of selective means by which EVs with protumor activity can be targeted is essential to provide a novel method for blood cancer therapy.

### 3.2. EVs for Drug Delivery

The small size of EVs enables them to cross the blood-brain barrier; the high similarity between the EV membrane and cellular membrane makes the former immune-tolerable, and the architecture of EVs protects their cargo from the degradation action by nucleases and proteases [95, 96]. These three features have made EVs an attractive mean for drug delivery. The concept of using EVs as therapeutic delivery system evolves around the idea that therapeutic molecule—such as miR, protein, or chemotherapy—can be endogenously or exogenously enclosed in EVs and applied in vitro or in vivo to be delivered to the target tissue [97]. Parent cells can be loaded or forced to overexpress an antitumor molecule, resulting in high loading of such molecules in the biosynthesized EVs, which can be isolated following their secretion from the parent cells (endogenous loading) [98]. For the exogenous loading, EVs are first isolated and then loaded with anticancer molecules using various methods, such as electroporation [98]. CML blasts are known to highly express interleukin 3 receptor (IL3R) on their surface compared with normal hematopoietic stem cells; therefore, IL3R may serve as a biomarker to specifically target CML blasts [99, 100]. BCR-ABL is a fused protein with a persistent tyrosine kinase activity that underpins the growth of CML [101]. Bellavia et al. engineered exosomes to endogenously overexpress a fragment of IL3 on their surface and to be loaded with imatinib (tyrosine kinase inhibitor) or *BCR*-*ABL* siRNA [102]. The application of the engineered exosomes selectively inhibited the growth of CML blasts in vitro and in vivo [102]. The overexpressed fragment of IL3 on the exosomes facilitated the selective targeting of CML blasts that also overproduced IL3R. Another similar example was reported by Huang et al. where exosomes were endogenoulsy loaded with inhibitors of miR21 and were modified to carry aptamer AS1411 on their surface [103]. It was reported that miR21 plays important roles in the pathogenesis of different types of malignancies, including hematological cancer [104]. Nucleolin, which exhibit high affinity to aptamer AS1411, is highly expressed on the surface of blood cancer cells, making it a possible marker that could be utilized for targeted therapy [105]. Using the aforementioned exosomes, Huang et al. reported enhanced endocytosis of the exosomes into CML blast due to the surface aptamer AS1411 as well as apoptosis induction in the leukemic cells as a result of delivery of miR21 inhibitors [103]. Decreasing the expression of miR328 was reported to be essential for imatinib resistance in leukemia cells [106]. Interestingly, the same study reported that alkalized exosomes enclosing exogenous miR328 restored imatinib sensitivity of the leukemic cells. In lymphoma cells, the endocytosis of exosomes carrying siRNA of c-myc, which is an oncogene that is implicated in the pathogenesis of various kinds of cancer, was found to inhibit tumor growth [107]. Taken together, EVs appeared as promising drug vehicles; hence, additional investigations are required to confirm their clinical application in the context of hematologic neoplasms.

### 3.3. Areas for Improvement

In the past decade, a large number of studies investigated the potential of EVs, secreted from MSCs or other types of cells, in the treatment of blood cancer. Collectively, these studies demonstrated promising roles for the EVs in the therapy of hematologic neoplasms, allo-HSCT, and/or Allo-HSCT-induced GVHD (summary is shown in Table 1). Despite the strikingly encouraging findings reported on the therapeutic potential of MSC-EVs, it is early for MSC-EVs to be considered off-shelf treatment for patients with blood neoplasms. In fact, several issues need to be addressed prior to the clinical application of MSC-EVs. For instance, the process of selecting a source of MSCs is still not clear; EVs from BM-MSCs have been more commonly studied compared with EVs from UC-MSCs in the context of the treatment of blood cancer. Therefore, additional investigations are required to compare the therapeutic potential of EVs from the two sources and provide guidelines for choosing the most potent source of MSC-EVs. Another important issue is the selection of suitable donor; age-matched donor-derived MSC-EVs exhibit variable immunomodulatory impact on allo-HSCT-induced GVHD [80]. Therefore, developing a standardize protocol for assessing the therapeutic potency of MSC-EVs prior to clinical application is essential to achieve desired treatment outcomes. Interestingly, MSC-EVs from younger donors appeared to have greater anticancer activity compared with those isolated from older healthy subjects [54]. Further work is needed to determine the optimum donor age that gives the most potent MSC-EVs.

Another critical point is the isolation procedure of MSC-EVs; researchers have been applying different protocols to extract MSC-EVs [108]. Commonly, ultracentrifugation has been deputed for the isolation of EVs. However, this technique is time-consuming, laborious, and prone to cosedimentation of macromolecules with EVs, reducing the purity of EVs [109]. Ultracentrifugation also has low EV recovery, and the ultra-gravity forces may disrupt the integrity of EVs. Collectively, these drawbacks of ultracentrifugation-based isolation may negatively impact the functionality of isolated EVs [109]. On the other hand, ultrafiltration followed by size exclusion chromatography (UF-SEC) was shown to reward higher EV yield with greater purity [110]. Furthermore, UF-SEC preserved the EV content of protein and other biomolecules, which positively impact the functionality of EVs [110]. Work is still ongoing to produce a golden standard method for EV isolation with optimal recovery, purity, and preserved integrity.

EVs encompass MVs and exosomes that display functional differences, due to the variation in their molecular content [111]. Development of a robust protocol that distinguishes between MVs and exosomes (as some population of MVs overlaps in size with exosomes) is needed in order to attribute the therapeutic potential to either MVs or exosomes, but not EVs. Durcin et al. reported protein and lipid signatures that differentiate between EVs with different sizes [112]. Such knowledge can be employed to create a standardized method based on affinity-dependent capture that isolates highly pure MVs or exosomes.

In vitro expansion of MSCs is dependent on cell culture format and condition. For example, the three-dimensional (3D) cell culture is more potent than the 2D cell culture in mimicking the physiological condition. Therefore, the 3D cell culture-based expansion of MSCs was shown to better preserve their physiological properties and enhance their therapeutic potential [113]. Similarly, cell culture cytokines and growth factors have been shown to potentize the therapeutic impact of MSC-EVs [114]. Therefore, procedure for in vitro amplification and pretreatment of MSCs remains to be standardized.

Other areas that have not been fully explored and standardized for the clinical use of MSC-EVs include potency criteria and storage of MSC-EVS [16]. There is a need for developing biomarkers that recognize the most potent and stable MSC-EVs within molecularly heterogeneous populations of MSC-EVs. Such biomarkers (especially surface proteins) can be then utilized to affinity-based isolate the best MSCs-EV for clinical application and long-term storage.

## 4. Conclusion

A great amount of research has been conducted in the past decade to study the biogenesis, composition, biological function, and therapeutic potential of MSC-EVs. These studies have provided valuable insights into the capability of MSC-EVs to exert antitumor and immunomodulatory effects. Therefore, MSC-EVs have been proposed as a promising option of therapy in patients (i) having hematological tumors, (ii) undergoing allo-HSCT, and/or (iii) developing GVHD postallo-HSCT. Despite the strikingly encouraging findings of the EVs, their clinical application remained hampered by the lack of sufficient preclinical studies and standardized protocols. Therefore, the future work should be directed to these two areas in order to clinically harness the MSC-EVs for the treatment of blood cancer.

## Figures and Tables

**Table 1:** Summary of the studies that investigated the potential of EVs secreted from MSCs and other kind of cells in the treatment of blood cancer.

Category	Source of EVs	Type of EVs	Disease	Experimental settings	Findings	Ref
Normal MSC-EV-based therapy	BM-MSCs from healthy donors and MM patients	Exosomes	MM	Mice were administrated with MM cells (MM.1S cell line) and exosomes from normal BM-MSCs or MM BM-MCSs. Next, differences in the exosomes content from the two sources were sought.	Unlike MM BM-MCS-exosomes, normal BM-MSC-exosomes inhibited the growth and metastasis of MM cells in mice. Normal BM-MSC-exosomes had low levels of IL6, CCL2, and fibronectin and high levels of 15a miR compared with MM BM-MSC-exosomes	[49]
Normal MSC-EV-based therapy	BM-MSCs from healthy donors and MM patients	MVs	MM	Normal or MM BM-MSC-MVs were in vitro incubated with MM cells (cell lines: U266, ARP1, MM1S, OPM2, and RPMI 8226). Next, the crosstalk between the MVs and MM cells was studied.	Normal BM-MSC-MVs inhibited the growth, survival, and migration of MM cells. In contrast to MVs from MM BM-MSCs, these from normal BM-MSC limited expression and/or phosphorylation of MAPKs (ERK1/2 and JNK), translation initiation (TI) factors (eIF4GI and eIF4E), TI regulator (TOR, MNK1/2 and 4EBP), and oncogenes (NF*κ*B, SMAD5, cyclin D, HIF1*α*, cMyc) in MM cells	[50]
Normal MSC-EVs-based therapy	BM-MSCs from healthy young and old donors	Exosomes	MM	Mice were inoculated with MM cells and BM-MSC-exosomes	MM-induced angiogenesis was strongly inhibited by BM-MSC-exosomes from young donors compared with that from old donors	[54]
Normal MSC-EV-based therapy	BM-MSCs from healthy donors and MDS patients	EVs	MDS	BM-MSC-EVs were in vitro incubated with hematopoietic progenitor cells CD34^+^.	Increased survival and clongeneity of hematopoietic progenitor were observed postincubation with BM-MSC-EVs from MDS patients but not healthy donors. Unlike MDS BM-MSC-EVs, these from healthy donors did not increase the expression of miR10a and miR 15a, which are implicated in the development of MDS, in the hematopoietic progenitor.	[56]
Normal MSC-EV-based therapy	BM-MSCs from healthy donors and AML patients	Exosomes	AML	BM-MSC-exosomes were in vitro incubated with AML cells.	In contrast to exosomes from AML patients, exosomes of healthy donors sensitized AML cells to chemotherapy (FLT3 inhibitor).	[60]
Normal MSC-EV-based therapy	BM-MSCs from healthy donors	EVs	Allo-HSCT-induced GVHD	BM-MSC-EVs were in vitro incubated with UCHSCs. Next, the transcriptome of UCHSCs was characterized postincubation. The BM-MSC-EV content of miRs was also sequenced. In mice, UCHSCs with or without BM-MSC-EVs were injected.	In vitro, BM-MSC-EVs supported the viability and restricted the differentiation of UCHSCs. Postincubation, UCHSCs increased the expression of genes involved in cell movement and growth and reduced expression of genes implicated in apoptosis. The EVs enclosed miRs whose target genes in UCHSCs were reduced implying a mechanism of action. Coadministration of UCHSCs with BM-MSC-EVs in mice augmented the engraftment of UCHSCs.	[75]
Normal MSC-EV-based therapy	BM-MSCs from healthy donors	Exosomes	Allo-HSCT-induced acute GVHD	Proinflammatory and anti-inflammatory cytokines were measured in BM-MSC-exosomes derived from four donors. The most potent source was selected for administration into patient with steroid-resistant acute GVHD postallo-HSCT. Proinflammatory cytokines of the patient's PBMCs were measured following administration of BM-MSC-exosomes.	BM-MSC-exosomes were found to carry reduced levels of proinflammatory cytokines (IL6, IL17a, IL21, TNF-a, IFN-g) and high levels of anti- inflammatory molecules (IL10, TGF beta, and HLAG). The immunomodulatory potential of the BM-MSC-exosomes differed significantly between the donors. The administration of BM-MSC-exosomes into the patient reduced the production of proinflammatory cytokines from PBMCs and improved the symptoms of GVHD.	[80]
Normal MSC-EVs-based therapy	BM-MSCs and NHDFs from healthy donors	Exosomes	Acute GVHD	Exosomes from BM-MSCs and NHDFs were administrated into mice with acute GVHD. Next, cellular immune response was studied. Exosomal content of miRs was also characterized.	Only exosomes from BM-MSCs were able to ameliorate the symptoms of GVHD. BM-MSC-exosomes suppressed T-helper cells and T-cytotoxic cells. In contrast to exosomes from NHDFs, these from BM-MSCs enclosed higher levels of miRs that target genes implicated in cellular proliferation, TCR signaling and GVHD.	[76]
Normal MSC-EV-based therapy	UC-MSC from healthy donors	EVs	Allo-HSCT-induced acute GVHD	UC-MSC-EVs were injected into a mouse model of allo-HSCT with acute GVHD. Next, the immune response in mice was studied.	UC-MSC-EVs alleviated the burden of acute GVHD and increased the survival rate of recipient mice. The serum levels of IL-2, TNF-*α*, and IFN-*γ* dropped postinjection with UC-MSC-EVs. In contrast, the level of IL10 in serum and the ration of Th-cells/T-cytotoxic cells were increased.	[77]
Normal MSC-EV-based therapy	BM-MSCs and fibroblasts from healthy donors	Exosomes	Chronic GVHD	Exosomes from BM-MSCs or fibroblasts were inoculated into mice with chronic GVHD. Next, the immune response in mice was studied.	Improvement of GVHD symptoms was possible only postinjection with BM-MSC-exosomes. The activation, migration, and infiltration of CD4 T-cells were inhibited by BM-MSC-exosomes but not fibroblast-exosomes. BM-MSC-exosomes also reduced the production of proinflammatory cytokines (IL-17A, IL-21, IL-22, and IL-2) in PBMCs of mice.	[78]
Exosome-based vaccine	AML cells (NP4 cell line)	Exosomes	AML	AML cell exosomes were in vitro incorporated into pulsed dendritic cells (DC). The DCs were then incubated with T-lymphocytes (CTLs). Next, CTLs as effector cells were in vitro incubated with AML cells.	CTLs became immunized against AML cells postincubation with DCs, in which AML cell exosomes were internalized. CTLs confer cytotoxicity against AML cells.	[82]
Exosome-based vaccine	CML cells (K562 cell line)	Exosomes	CML	In vitro pulsed DCs uptook CML cell exosomes. CTLs were incubated with the DCs. Next, CML cells were treated with the CTLs in vitro and in vivo.	DCs incorporating CML cell-exosome-induced immunity of CTLs that caused death of CML cells in vitro. The administration of the DCs to mice protected them from developing CML postinoculation with CML cells.	[83]
Exosome-based vaccine	LL cells (L1210 cell line)	Exosomes	LL	Exosomes from LL cells that lack or express TGF-*β*1 were independently internalized by DCs. Next, DCs were injected into mice. The mice were then inoculated with LL cells.	The DCs incorporated with exosomes lacking the expression of TGF-*β*1 conferred stronger protection against LL compared with DCs that uptook TGF-*β*1 expressing exosomes.	[87]
Targeting EVs	BM-MSCs (HS-5 cell line)	EVs	AML and CML	FGFR was inhibited (or gene silenced) in BM-MSCs (HS-5 cell line). Next, the BM-MSCs were in vitro incubated with AML and CML cells (MOLM14 and K562 cell lines). The leukemic cells were also administrated to *Fgf2* -/- and *Fgf2* +/+ mice.	Targeting FGFR inhibited the release of exosomes from BM-MSC, which abolished the protective impact of the BM-MSCs on the leukemia cells. Mice lacking the expression of FGF2 survived longer than those that that expressed FGF2.	[89]
Targeting EVs	Lymphoma cells (EL4 cell line)	Exosomes	Lymphoma	Mice were injected with lymphoma cells (EL4 cell line). Then, they were treated with cyclophosphamide with or without DMA.	DMA inhibited exosomes release in vivo. Furthermore, the combination of cyclophosphamide with DMA significantly reduced the tumor growth, while cyclophosphamide alone had a little effect on the tumor growth.	[90]
Targeting EVs	MM cell lines (RPMI8226, H929, MM1S and U266)	Exosomes	MM	Exosomes from MM cell lines (RPMI8226, H929, MM1S and U266) with or without endocytosis inhibitor (heparin) were incubated with BM-MSCs.	In the absence of heparin, MM cell exosomes were internalized by BM-MSCs, which reprogrammed the latter to support the MM growth. Heparin inhibited the uptake of MM cell exosomes by BM-MScs; hence, the tumor-supportive impact of BM-MSCs was abolished.	[92]
Targeting EVs	AML stem cells	Exosomes	AML	AML stem cells were transfected with miR-34c-5p mimic, and the proliferation of AML cells was assayed. Mice were transplanted with AML cells. Three weeks later, the mice were treated with miR-34c-5p agomir.	In vitro, the increased expression miR-34c- blocked the release of exosomes from AML stem cells. In vivo miR-34c-p-dependent blockage of exosomes shedding antagonized the growth of AML in mice.	[93]
Targeting EVs	B-cell lymphoma primary cells and cell lines (Su-DHL-4, Balm-3, OCI-Ly1).	Exosomes	B-cell lymphoma	Surface expression of CD20 was measured on exosomes from B-cell lymphoma primary cells and cell lines (Su-DHL-4, Balm-3, OCI-Ly1). These exosomes were in vitro incubated with known concentration of anti-CD20 antibody (rituximab). Plasma from patients receiving rituximab was also incubated with the exosomes. B-cell lymphoma cells were treated with inhibitor of exosome release and then were treated with rituximab.	B-cell lymphoma cells express high level of surface CD20, which bound to rituximab, protecting the malignant cells from the cytolytic effect of the drug. Inhibition of exosomes release form B-cell lymphoma cells sensitized them to rituximab.	[94]
EVs for drug delivery	Embryonic kidney cells (HEK293T-cell line)	Exosomes	CML	HEK293T cell were forced to express surface IL3 and were treated with a kinase inhibitor (Imatinib) or BCR-ABL siRNA. Next, Exosomes with the surface expression of IL3 that contained imatinib or BCR-ABL siRNA were isolated and applied to imatinib-resistant CML blast (LAMA84 and K562 cell lines) in vitro. The engineered exosomes were also injected into mice that were previously inoculated with CML blasts.	The engineered exosomes selectively inhibited the growth of CML in vitro and in vivo. The distribution analysis of the engineered exosomes in vivo showed that they accumulated at the tumor site.	[102]
EVs for drug delivery	Embryonic kidney cells (HEK293T-cell line)	Exosomes	CML	HEK293T cells were transfected to with plasmid of BFP-miR-21 sponge-MS2. Exosomes released from the engineered HEK293T cells were isolated and modified to carry aptamer AS1411 on their surface. The engineered exosomes were applied in vitro to CML blasts (K562 cell line).	The exosomes were u-taken by the CML blast, and miR-21 sponge-MS2 was delivered, leading to apoptosis induction in the malignant cells. Aptamer AS1411 enhanced the ability of the exosomes to target CML blasts.	[103]
EVs for drug delivery	Embryonic kidney cells (HEK293T-cell line)	Exosomes	CML	Exosomes released from HEK293T cells were isolated, alkalinized, and exogenously loaded with miR328. The exosomes were used for the treatment of imatinib-resistant CML blasts (K562 cell line).	The endocytosis of the exosomes into imatinib-resistant CML blasts sensitized the later to imatinib.	[106]
EVs for drug delivery	Fibroblasts (NIH3T3 cell line)	Exosomes	Lymphoma	Exosomes from NIH3T3 cells were either endogenously or exogenously loaded with siRNA of c-myc. Next, lymphoma cells with an overexpression of c-myc were treated with the exosomes.	The delivery of the exosomes into lymphoma cells reduced the expression of c-myc and inhibited the growth of lymphoma.	[107]

EVs: extracellular vesicles; MSC: mesenchymal stem cells MM: multiple myeloma; MV: microvesicles; MDS: myelodysplastic syndrome; AML: acute myeloid leukemia; HSCT: hematopoietic stem cell transplantation; GVHD: graft versus host disease; LL: lymphoid leukemia; CML: chronic myeloid leukemia.

## Data Availability

No data availability statement was included in the manuscript.
